# What Amount of Weight Loss Can Entail Anorexia Nervosa or Atypical Anorexia Nervosa After Bariatric Surgery?

**DOI:** 10.1002/eat.24286

**Published:** 2024-09-05

**Authors:** Johannes Hebebrand, Jochen Antel, Eva Conceição, Abigail Matthews, Anke Hinney, Triinu Peters

**Affiliations:** ^1^ Department of Child and Adolescent Psychiatry, Psychotherapy and Psychosomatics, University Hospital Essen (AöR) University of Duisburg‐Essen Essen Germany; ^2^ Center for Translational Neuro‐ and Behavioral Sciences, University Hospital Essen University of Duisburg‐Essen Essen Germany; ^3^ Faculty of Psychology and Education Sciences University of Porto Porto Portugal; ^4^ Department of Psychiatry and Psychology Mayo Clinic Rochester Minnesota USA; ^5^ Section of Molecular Genetics in Mental Disorders University Hospital Essen Essen Germany; ^6^ Institute of Sex and Gender‐Sensitive Medicine University Hospital Essen Essen Germany

**Keywords:** anorexia nervosa, atypical anorexia nervosa, bariatric surgery, premorbid BMI, pre‐operative BMI, weight loss

## Abstract

**Objective:**

Post‐operative development of restrictive eating disorders can occur in patients after bariatric surgery. In children and adolescents with anorexia nervosa (AN) or atypical AN, premorbid body mass index (BMI) has recently been shown to predict total weight loss. We hypothesized that pre‐operative BMI similarly predicts weight loss and the development of a restrictive eating disorder in adult bariatric patients.

**Method:**

A PubMed search identified case studies/series of 29 adult females who developed AN or atypical AN/eating disorder not otherwise specified following bariatric surgery. Non‐parametric Spearman's correlation (*r*_*s*_) between pre‐operative BMI and total weight loss was calculated; a scatterplot was used to illustrate the relationship between pre‐operative/premorbid BMI and weight loss in kg for 29 bariatric patients and 460 children and adolescents with AN or atypical AN as published previously.

**Results:**

The correlation between pre‐operative BMI and weight loss among bariatric patients was *r*_*s*_ = 0.65 (*p* = 0.0001). Scatterplot data of this relationship fit the previously identified pattern in children and adolescents with AN or atypical AN.

**Discussion:**

The prediction of weight loss by pre‐operative/premorbid BMI appears applicable across the weight spectrum, from underweight to severe obesity, thus strengthening our hypothesis of underlying regulatory mechanisms for the development of AN and atypical AN. Such data may guide the determination of critical weight loss thresholds that trigger eating disorder development in predisposed individuals.

**Summary**

- In children and adolescents with anorexia nervosa (AN) or atypical AN, premorbid body mass index (BMI) consistently predicts the amount of weight loss in kilograms (kg) before admission for inpatient treatment.
- The current study, based on 29 adults who developed a restrictive eating disorder following bariatric surgery in previously published case reports/series, examined the relationship between pre‐operative BMI and the amount of weight loss in kg before diagnosis.
- Indeed, bariatric patients with post‐operative AN/atypical AN lost substantially *more* weight than children and adolescents with AN/atypical AN in a registry‐based sample; pre‐operative BMIs among bariatric patients were also higher than premorbid BMIs among the children and adolescents.
- We conclude that pre‐operative/premorbid BMI predicts weight loss in patients who develop restrictive eating disorders.
- Accordingly, future research may guide the delineation of critical weight loss thresholds in relationship to pre‐operative/premorbid BMI upon which the risk for development of a restrictive eating disorder sets in.

## 1 Introduction

Premorbid body mass index (BMI), BMI‐centile‐for‐age, and BMI‐standard deviation score predict total weight loss among hospitalized female children and adolescents with anorexia nervosa (AN) or atypical AN with Spearman rank correlation coefficients exceeding *r*_*s*_ = 0.85 (Coners, Remschmidt, and Hebebrand 1999; Hebebrand and Seitz, et al. 2024; Matthews et al. 2024). Accordingly, Hebebrand and Seitz, et al. (2024) proposed regulatory mechanisms underlying AN and atypical AN, whereby *weight loss* triggers the development of an AN‐like phenotype across the weight spectrum. Herein termed “entrapment,” characteristics include a persistent and impairing preoccupation with food and/or weight, reduced nutritional intake, and weight phobia in the context of psychological and physiological adaptations to starvation (e.g., Brode and Mitchell 2019; Hebebrand and Plieger, et al. 2024).

Among patients undergoing bariatric surgery, the development of post‐operative AN or atypical AN has repeatedly been described (e.g., Brode and Mitchell 2019; Conceicao and Orcutt, et al. 2013; Conceicao and Vaz, et al. 2013; Hilbert et al. 2022; Segal, Kinoshita Kussunoki, and Larino 2004) with atypical AN occurring more frequently than AN. In this population, distinguishing *disordered* eating from normative post‐operative eating is particularly complex because dietary restriction is prescribed to optimize post‐operative weight loss. Accordingly, positive surgical outcomes hinge upon adherence to rigid food rules that undoubtedly require a preoccupation with food and/or weight; body dissatisfaction and fears of (re)gaining weight are also common (Conceicao and Hilbert 2023; Hilbert et al. 2022; Ivezaj et al. 2021; Ramalho et al. 2015). Nevertheless, case studies have demonstrated that in single patients, post‐operative weight loss resulted in a BMI below the AN weight criterion; these cases also demonstrated psychopathological features of AN and physical symptoms of starvation (e.g., Atchison et al. 1998; Conceicao and Orcutt et al. 2013; Conceicao and Vaz, et al. 2013). Atypical AN (Watson, Riazi, and Ratcliffe 2020) or eating disorder not otherwise specified (EDNOS) per DSM‐IV (APA 1994) (e.g., Conceicaod and Orcutt, et al. 2013) have more commonly been described following bariatric surgery than AN. For example, in a German registry study of 748 bariatric patients, AN had not been diagnosed at any one of the six annual follow‐ups (Hilbert et al. 2022). In contrast, atypical AN diagnosed with clinical interview was observed in approximately 15% of patients attending the one‐ and two‐year follow‐ups and at rates above 8% at subsequent visits.

The current study aimed to assess whether previously demonstrated associations between premorbid BMI and weight loss in children and adolescents with AN or atypical AN (Coners, Remschmidt, and Hebebrand 1999; Hebebrand and Seitz, et al. 2024; Matthews et al. 2024) extend to adults with severe obesity who developed a restrictive eating disorder following bariatric surgery.

## 2 Method

PubMed searches, independently conducted by JH and AH (11/1/2023), identified 56 English‐written peer‐reviewed articles with keywords “anorexia nervosa” and “bariatric surgery.” Additional inclusion criteria were: (1) publication as case report or series; (2) cases of patients of female sex‐at‐birth who developed AN or atypical AN (or EDNOS) following bariatric surgery‐induced weight loss; and (3) reported pre‐operative BMI (or pre‐operative weight and height) *and* total weight loss (in kg or kg/m^2^). Nine articles fulfilled these inclusion criteria (Atchison et al. 1998; Conceicao and Orcutt, et al. 2013; Guisado et al. 2002; Scioscia et al. 1999; Segal, Kinoshita Kussunoki, and Larino 2004; Shear and DeFilippis 2015; Tortorella et al. 2015; Watson, Riazi, and Ratcliffe 2020; Williams et al. 2020). An additional case series that met study inclusion criteria (Conceicao and Vaz, et al. 2013), but not listed in PubMed, was provided by EC. Data extraction was performed by JH and independently by AH and JA; discrepancies were settled between JH, JA, and TP. Eight of the ten studies enabled determination of both patients' pre‐operative BMI and weight loss in kg defined by the difference between pre‐operative and presenting weight. Two studies Conceicao and Orcutt, et al. (2013) and Segal, Kinoshita Kussunoki, and Larino (2004) provided weight loss in kg/m^2^ without adult height; corresponding authors were contacted to obtain patients' body heights to calculate weight loss in kg. These data were available for one study (Conceicao and Orcutt, et al. 2013). For the remaining study (Segal, Kinoshita Kussunoki, and Larino 2004) of five Brazilian patients, the average body height of females in Brazil (159 cm; https://en.wikipedia.org/wiki/Average_human_height_by_country) was used to estimate weight loss in kg. For all cases, weight loss was calculated from the lowest reported post‐operative weight or BMI (which was identical to the presenting weight/BMI if no prior lower weight/BMI was reported).

The ten studies included data of 32 adult patients, 29 of whom had developed a restrictive eating disorder (for investigator‐based diagnoses see Table 1) after post‐operative weight loss; an additional three patients developed bulimia nervosa (BN) (see Conceicao and Orcutt, et al. 2013; Conceicao and Vaz, et al. 2013 in Table 1). For the current study and in line with DSM‐5 (American Psychiatric Association 2013), BMI < or ≥18.5 kg/m^2^ was used to reclassify the 29 patients with restrictive eating disorders into AN or atypical AN.

**TABLE 1:** Extracted data sets of 32 adult patients identified in 10 published case reports/series who developed an eating disorder after bariatric surgery.

Study	Patients	Age (years)	Pre‐operative BMI (kg/m^2^)	Pre‐operative weight (kg)	Weight loss in kg	Weight loss in %	Weight loss in kg/m^2^	Lowest reported post‐operative BMI (kg/m^2^)	Height (m)	Diagnosis according to publication	Elapsed time between surgery and presentation (months)	Surgical procedure
Atchison et al. (1998)	A	44	36.8	118	57	48.3	17.8	19.0	1.79^b^	AN	12	A: gastric bypass
	B^a^	53	48.6	136	94	69.1	33.7	15.0	1.67^b^	AN	17	B: gastric reduction (operated twice)
Scioscia et al. (1999)	A	38	56	138	98.7	71.5	39.8	16.2	1.58^c^	AN‐B/P	24	Gastric bypass
Guisado et al. (2002)	A	32	43	125	55	44.0	18.8	24.2	1.70	EDNOS	n.r.	Gastroplasty
Segal, Kinoshita Kussunoki, and Larino (2004)	A	42	48	121.3	60.3	49.9	24	24	1.59^d^	EDNOS	42	Gastric bypass (Capella)
	B	41	52	131.5	80.4	61.4	32	20	1.59		48	
	C	44	48	121.3	60.3	49.9	24	24	1.59		36	
	D	42	57	144.1	67.8	47.2	27	30	1.59		24	
	E	51	46	116.3	40.1	34.6	16	30	1.59		36	
Conceicao and Orcutt, et al. (2013)	A^e^	65	58.6	136.1	80.8	40.6	34.8	23.8	1.52	EDNOS	3 months to 26 years (M = 5.7 years; SD = 7.8)	Roux‐en‐Y (*n* = 9); Duodenal switch (*n* = 1); Gastric band (*n* = 2)
	B	52	79.4	203.2	116.5	42.7	45.5	33.9	1.60	EDNOS		
	C	31	48.7	141.1	55.3	60.8	19.1	29.6	1.70	EDNOS		
	D	52	42.4	112.7	66.5	59.0	25.0	17.4	1.63	AN‐R		
	E	64	47.4	129.3	71.4	44.7	26.2	21.2	1.65	EDNOS		
	F	23	66.2	169.5	68.1	40.2	26.6	39.6	1.60	BN		
	G	69	43.1	111.7	37.0	66.9	15.2	28.8	1.61	EDNOS		
	H	29	43.6	133.8	48.2	64.0	15.7	27.9	1.75	BN		
	I	61	40.6	124.7	60.1	48.1	20.1	21.1	1.75	EDNOS		
	J	52	47.4	129.3	93.4	27.7	34.3	13.1	1.65	AN‐B/P		
	K	26	44.8	111.1	68.5	38.3	27.6	17.3	1.57	AN‐R		
	L	38	45.2	109.3	61.4	43.8	25.4	19.8	1.56	EDNOS		
Conceicao and Vaz, et al. (2013)	A	40	46.0	130.0	70	53.8	24.8	21	1.68	AN^f^	18	Gastric sleeve
	B	31	41.0	109	49	45.0	18.4	21	1.63	AN^f^	24	Gastric sleeve
	C	45	49.9	120	50	41.7	20.8	29.1	1.55	BN	Second post‐operative year	Gastric bypass
Tortorella et al. (2015)	A	29	46.0	103.6	66.6	64	29.6	16.4	1.50^b^	AN	“a few months” after 24‐month follow‐up”	Bilio‐intestinal bypass
Shear and DeFilippis (2015)^g^	A	52	55.5	147.7	100.9	68.3	37.9	17.6	1.63	AN	48	Roux‐en‐Y
Watson, Riazi, and Ratcliffe (2020)^h^	A	31	53.4	143.6	59	41.1	21.7	31.7	1.64	OSFED —Atypical AN	96	Roux‐en‐Y
	B	29	47.1	140.9	52	33.4	17.4	29.7	1.73		12	Roux‐en‐Y
	C	35	46.4	103.0	38.5	45	17.3	29.1	1.49		9.5	Roux‐en‐Y
	D	38	52.6	161.0	83.5	51.9	27.3	25.3	1.75		36	Roux‐en‐Y
	E	55	42.2	104.0	28.8	27.7	11.7	30.5	1.57		24	Roux‐en‐Y
Williams et al. (2020)	A	18	43	121.1^h^	46.3	38.2	16.4	23.6	1.68	AN‐R	11	Sleeve gastrectomy

Abbreviations: AN, Anorexia Nervosa; AN‐B/P, Anorexia Nervosa, binge‐eating/purging type; AN‐R, Anorexia Nervosa, restricting type; BN, Bulimia Nervosa; EDNOS, Eating Disorder Not Otherwise Specified; n.r., not reported; OSFED, Other Specified Feeding or Eating Disorder.

^a^ Second bariatric surgery, data not available for first surgery.

^b^ Height deduced based on delineated weight in kg and BMI at one time point.

^c^ Height in feet and inches converted to meters.

^d^ Average height of a Brazilian female (https://en.wikipedia.org/wiki/Average_human_height_by_country).

^e^ Patients A‐L correspond to 1–12 in manuscript.

^f^ Specification of the authors: AN but weight criterion not fulfilled.

^g^ The patient had had AN prior to development of extreme obesity.

^h^ Pre‐operative weight loss of 10.2 kg during her pre‐operative course (12 months) included in total weight loss; initial BMI 43.

### 2.1 Statistical Analyses

Descriptive means and standard deviations (SD) were determined. Non‐parametric Spearman's correlation coefficients (*r*_*s*_) were calculated between pre‐operative BMI and weight loss in kg for all 29 patients with a restrictive eating disorder and descriptively for patients with AN (*n* = 7) and atypical AN (*n* = 22). Exact two‐sided significance was calculated with alpha = 0.05. Further, the relationship between pre‐operative BMI and weight loss was assessed within the previously published scatterplot of the relationship between premorbid BMI and weight loss in 460 hospitalized children and adolescents with AN (*n* = 411) or atypical AN (*n* = 49) from the German Registry for Children and Adolescents with AN (Hebebrand and Seitz, et al. 2024). Given the inference of average height (159 cm) for the five Brazilian cases (Segal, Kinoshita Kussunoki, and Larino 2004), we increased and decreased the heights of these patients by 1 SD (6.5 cm in Brazilian females; see Silva et al. 2010) and used a z‐test to compare resultant correlation coefficients. Analyses were performed using IBM SPSS Statistics 29.0.0 for Windows.

## 3 Results

**FIGURE 1:**
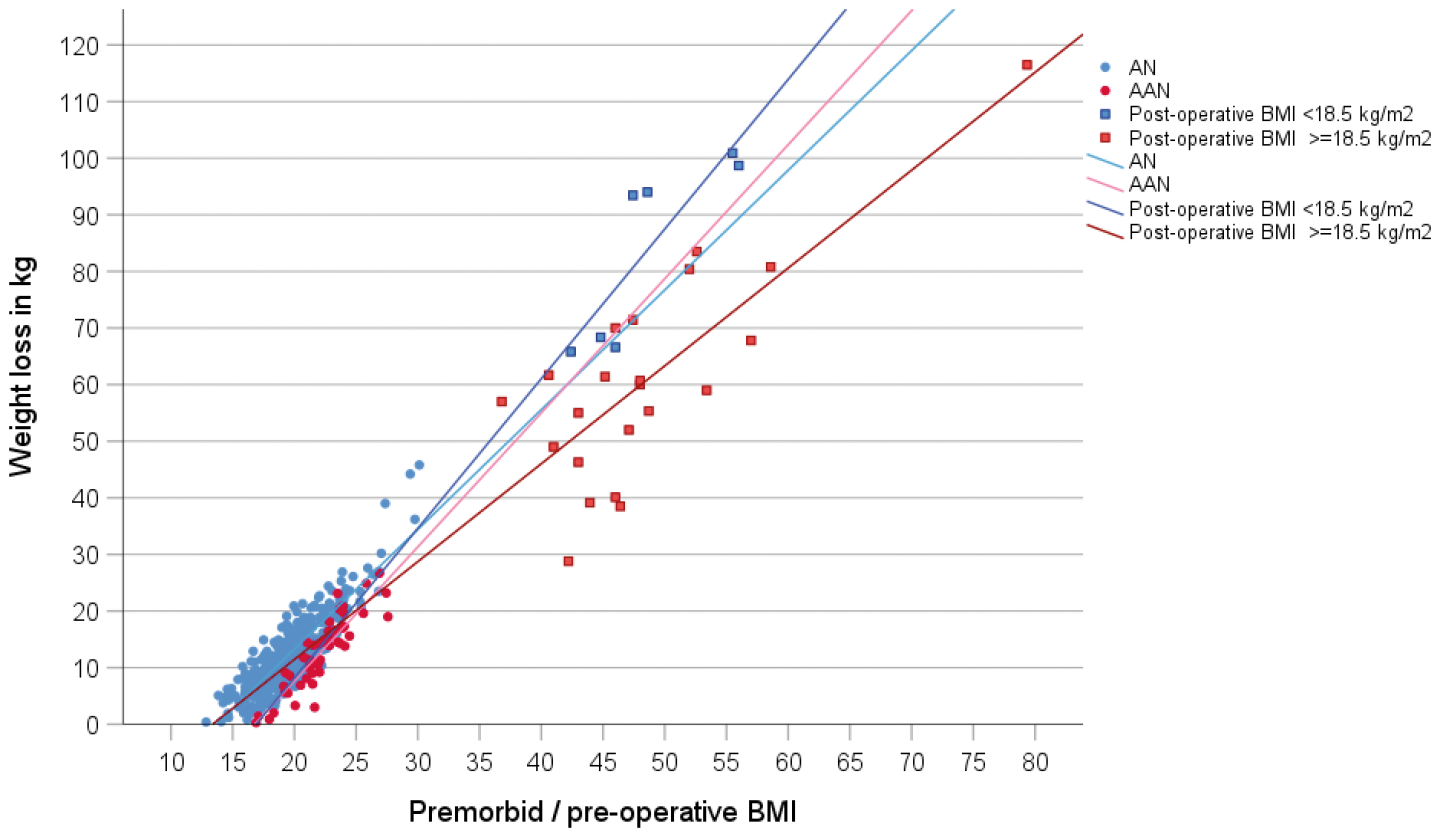
Scatterplot of the relationship between weight loss in kg and premorbid BMI for adolescent inpatients with anorexia nervosa (AN) (*n* = 411) or atypical AN (*n* = 49) from the German Registry for Children and Adolescents with Anorexia Nervosa (Hebebrand and Seitz, et al. 2024), and pre‐operative BMI in 29 identified adult bariatric patients who developed AN or atypical AN. Regression lines with *R* ^2^: AN = 0.77, atypical AN = 0.80; post‐operative BMI <18.5 = 0.72; post‐operative BMI ≥18.5 = 0.63.

Means for age, pre‐operative BMI, weight loss, and percent weight loss of the 29 patients who developed restrictive eating disorders after bariatric surgery were 43.3 years (SD = 12.8), 48.5 kg/m^2^ (SD = 7.9), 66.2 kg (SD = 20.8), and 48.9% (SD = 12.1), respectively (see Table 1 for extracted data sets). Patients in the largest BMI‐subgroup (40.0–49.9 kg/m^2^; *n* = 20) had a mean pre‐operative BMI of 45.2 kg/m^2^ (SD = 2.6) and a mean weight loss of 58.7 kg (SD = 16.9). Figure 1 extends the previously published relationship between premorbid BMI and weight loss for pediatric inpatients (Hebebrand and Seitz, et al. 2024) to the 29 adult bariatric patients. Considering both samples, patients' ages ranged from 9.8 to 65 years (Table 1); premorbid (children/adolescents)/pre‐operative (adults) BMI ranged from 13 to 79 kg/m^2^ and weight loss from 0.4 to 116.5 kg (Figure 1). The seven bariatric patients with AN lost the most weight for any given pre‐operative BMI. Spearman's correlation between pre‐operative BMI and total weight loss (kg) among the 29 bariatric patients with our diagnoses of AN or atypical AN was *r*_*s*_ = 0.65 (*p* = 0.0001; for correlations for patients with AN and atypical AN see legend to Figure 1). Systematic increments and decrements of the heights of the five Brazilian patients by 6.5 cm resulted in correlations of *r*_*s*_ = 0.45 (*p* = 0.01) and *r*_*s*_ = 0.40 (*p* = 0.03), respectively. Z‐tests did not demonstrate significant differences between correlation coefficients obtained when using mean height for the five Brazilian patients and mean height + 1 SD (*p* = 0.20) or mean height−1 SD (*p* = 0.29).

## 4 Discussion

Consistent with our hypothesis and prior research in children and adolescents with AN or atypical AN (Coners, Remschmidt, and Hebebrand 1999; Hebebrand and Seitz, et al. 2024; Matthews et al. 2024), pre‐operative BMI was significantly correlated with weight loss in adult bariatric patients. Upon joint assessment with Hebebrand and Seitz, et al.'s (2024) pediatric sample, pre‐operative/premorbid BMI, with values ranging from categorical underweight to severe obesity, was systematically associated with weight loss. As discussed among the pediatric sample (Hebebrand and Seitz, et al. 2024), using a BMI cut‐off of 18.5 kg/m^2^ to differentiate diagnoses of AN or atypical AN in adult bariatric patients with an AN‐like phenotype seemingly splits an otherwise homogenous sample. We concur with Golden and Walsh (2024) that current evidence supports conceptualization of AN and atypical AN as part of a spectrum‐based restrictive eating disorder (Golden and Walsh 2024).

These results further substantiate our hypothesis of underlying regulatory mechanisms in restrictive eating disorders, in which premorbid or pre‐operative (in bariatric patients) weight, adjusted for height, predetermines the amount of weight loss required for the emergence of entrapment. It is important to note, however, that among the 29 patients in our sample, the amount of weight loss that triggered entrapment cannot be deduced from the amount of weight loss demonstrated at diagnosis. Weight loss may have persisted *after* the onset of entrapment before the referral visit. Thus, in our sample, the amount of weight loss required for entrapment is likely lower than that depicted in Figure 1. Indeed, in single cases the post‐operative onset of AN‐like symptomatology occurred prior to attainment of the lowest weight (e.g., Guisado et al. 2002; Scioscia et al. 1999); in addition, one case report refers to a patient who had AN prior to developing extreme obesity (Shear and DeFilippis 2015). Accordingly, we hypothesize that our admission data merely approximates the relationship between pre‐operative/premorbid BMI and the amount of weight loss required for entrapment. Further research is warranted to potentially identify critical weight thresholds that entail entrapment risk in predisposed females across the weight spectrum and their relationship to the amount of weight loss at referral. Research should additionally address the underlying mechanisms.

Based on our initial data, an adult patient with a pre‐operative BMI of 50 kg/m^2^ who develops a post‐operative restrictive eating disorder would lose more than 40 kg before referral (Figure 1). In contrast, in a 12‐year‐old girl with a premorbid BMI of 16 kg/m^2^ (e.g., premorbid weight: 38.5 kg, height: 1.55 m, 13th BMI‐centile‐for‐age) (Neuhauser et al. 2013) weight loss before referral may be minimal (< 2 kg). Further studies and meta‐analyses are required to ensure the generalizability and absolute values of such thresholds.

Such knowledge may prove beneficial from both a clinical and preventative perspective. Thus, in bariatric patients, attaining weight loss that corresponds to the *lower* limit of the range entailing entrapment could alert patients and physicians to this potential danger. The relative weight loss (*M* = 48.3%) of the 29 bariatric patients was substantially higher than that reported (approximate *M* = 30%) one‐year following Roux‐en‐Y, gastric sleeve, or anastomosis gastric bypass in a large and international data set of the Federation for the Surgery of Obesity and Metabolic Disorders (Welbourn et al. 2019). In that study, 73.7% of bariatric patients were female; the mean pre‐operative BMI was 41.7 kg/m^2^ (Welbourn et al. 2019) and thus almost 7 kg/m^2^ lower than in the 29 bariatric patients with AN or atypical AN. While a direct comparison with Welbourn et al.'s (2019) bariatric sample is not possible, our bariatric cases who developed post‐operative entrapment seemingly demonstrated substantially greater weight loss upon adjustment for pre‐operative BMI. This assumption is underscored upon comparison with a study of 1993 bariatric patients (1708 females; Ochner et al. 2013), which assessed average weight loss in relationship to pre‐operative BMI at 12, 18, 24, and 36 months after bariatric surgery. Mean weight loss exceeded 50 kg after 12, 18, and 24 months *only* among the subgroup of patients with the highest pre‐operative BMI (≥60 kg/m^2^). In the other patient subgroups, (pre‐operative BMI 35–39.9, 40–49.9, or 50–59.9 kg/m^2^) mean weight loss was less than 50 kg. For example, mean weight loss was approximately 40 kg for patients with a pre‐operative BMI between 40.0 and 49.9 kg/m^2^. In contrast, among the 29 bariatric patients who developed AN or atypical AN, with only two demonstrating a pre‐operative BMI ≥60 kg/m^2^, mean weight loss was 66.3 kg. For the large patient subgroup with a pre‐operative BMI between 40.0 and 49.9 kg/m^2^, mean weight loss was 57.9 kg. Accordingly, it is likely that the amount of weight loss demonstrated in bariatric patients who develop AN or atypical AN is substantially higher than that experienced in bariatric patients who do not incur a restrictive eating disorder (matched for pre‐operative BMI). This suggests that above average weight loss for any pre‐operative BMI should alert physicians to the risk for eating disorder development in bariatric patients.

The relationship between pre‐operative/premorbid BMI and weight loss should also facilitate a defined *upper* limit of weight loss that induces entrapment. Thus, the percentage of females who would *not* develop entrapment after surpassing the upper limit weight loss threshold is unknown. Consider an adolescent with a premorbid BMI of 25 kg/m^2^, who loses 30 kg of body weight *without* experiencing entrapment (form a vertical line at 25 kg/m^2^ in Figure 1). Despite substantial weight loss, she had not developed a restrictive eating disorder, potentially indicating the absence of a genetically and environmentally determined predisposition. A systematic comparison of pre‐operative BMI distributions of bariatric patients is warranted to determine the percentage of patients who develop a restrictive eating disorder at exceedingly higher weight loss cut‐offs. Overall, a thorough knowledge of the distribution of age‐dependent weight loss (intentional or unintentional) in relationship to pre‐operative/premorbid BMI is needed to understand the risk of weight‐loss induced entrapment.

Limitations of our study include: (i) In bariatric patients, pre‐operative weight is defined as a patient's most recently documented weight within 30‐days of surgery (Sun et al. 2020); we were unable to confirm this methodology for the 29 patients. Additionally, for some patients, immediate pre‐operative weight loss is recommended one to 3 weeks prior to surgery. Thus, our pre‐operative BMI data were not assessed systematically. (ii) For five patients from Brazil (Segal, Kinoshita Kussunoki, and Larino 2004) weight loss was inferred from average height; accordingly, the actual amount of weight loss may have been higher or lower. Nevertheless, the correlation coefficients between pre‐operative BMI and weight loss did not differ significantly in all 29 patients using pre‐operative BMI calculated by mean height and mean height +1 or −1 SD for the five patients from Brazil. (iii) Whereas pre‐operative BMI data for the bariatric patients reflected measured pre‐operative weight and height, premorbid BMIs in the pediatric sample (Hebebrand and Seitz, et al. 2024) were estimated based on recalled premorbid weight and measured admission height. (iv) The elapsed time between bariatric surgery and presentation substantially varied in the 29 adults, potentially increasing data skewness. (v) Our literature search was limited to PubMed. We were fortunate to include an additional study, provided by author EC (Conceicao and Vaz, et al. 2013).

The prevalence of patients who develop AN or atypical AN after bariatric surgery requires further study. Development and validation of eating disorder assessment measures among bariatric patients are necessary. Identifying critical weight loss thresholds that potentially induce entrapment in predisposed individuals could assist in differentiating bariatric patients at risk for developing post‐operative AN or atypical AN. Considering the identified methodological limitations, we speculate that the actual relationship between pre‐operative BMI and weight loss may be even stronger than detected in our analyses.

## Author Contributions

**Johannes Hebebrand:** conceptualization, data curation, methodology, resources, supervision, writing – original draft. **Jochen Antel:** conceptualization, data curation, writing – original draft. **Eva Conceição:** data curation. **Abigail Matthews:** writing – original draft. **Anke Hinney:** conceptualization, data curation. **Triinu Peters:** conceptualization, data curation, formal analysis, methodology, software, supervision, visualization.

## Conflicts of Interest

Johannes Hebebrand (JH), and Jochen Antel (JA) are named as inventors in patent applications of the University of Duisburg‐Essen (UDE) on the use of leptin analogues for the treatment of AN, atypical AN and depression. JH received speaker's honoraria from Amryt Pharmaceuticals and Novo Nordisk. Eva Conceição (EC) received speaker's honoraria from Novo Nordisk. All other authors declare that the research was conducted in the absence of any commercial or financial relationships that could be construed as a potential conflict of interest.

## Data Availability

Data sharing is not applicable to this article as no new data were created or analyzed in this study.
